# Assessment of Endothelial Function in Patients with COVID-19 Using Peripheral Arterial Tonometry

**DOI:** 10.3390/life14111512

**Published:** 2024-11-20

**Authors:** Athanasios Moulias, Rafail Koros, Angeliki Papageorgiou, Spyridon Katechis, Panagiotis Patrinos, Aikaterini Trigka-Vasilakopoulou, Athanasios Papageorgiou, Ourania Papaioannou, Karolina Akinosoglou, Georgios Leventopoulos, Grigorios Tsigkas, Argyrios Tzouvelekis, Periklis Davlouros

**Affiliations:** 1 Department of Cardiology, General University Hospital of Patras, 26504 Patras, Greece; dramoulias@live.com (A.M.); korosraf@hotmail.com (R.K.); patrpan@hotmail.com (P.P.); ktvasilak1@gmail.com (A.T.-V.); thanasispapag95@gmail.com (A.P.); levent2669@gmail.com (G.L.); gregtsig@gmail.com (G.T.); pdav@upatras.gr (P.D.); 2 Department of Inherited and Rare Cardiovascular Diseases, Onassis Cardiac Surgery Center, 17674 Athens, Greece; 3 Department of Rheumatology, General Hospital of Asklipieio Voulas, 16673 Athens, Greece; katechissp@gmail.com; 4 Department of Pneumonology, General University Hospital of Patras, 26504 Patras, Greece; ouraniapapaioannou@outlook.com (O.P.); atzouvelekis@upatras.gr (A.T.); 5 Department of Internal Medicine, General University Hospital of Patras, 26504 Patras, Greece; akin@upatras.gr

**Keywords:** COVID-19-induced endothelial dysfunction, EndoPAT, reactive hyperemia index in COVID-19 patients

## Abstract

There is increasing evidence that COVID-19 induces endothelial dysfunction that may precede thrombotic and cardiovascular complications. The aim of this study is to evaluate endothelial function using peripheral arterial tonometry (EndoPAT). The primary endpoint is the hyperemic vascular response index (LnRHI) at two months post-discharge. Secondary endpoints include the LnRHI during hospitalization and at six-month follow-up, the proportion of patients with endothelial dysfunction (LnRHI ≤ 0.51), and the incidence of thrombotic events, cardiovascular complications, and mortality during the follow-up period. The study included 23 COVID-19 patients and 22 COVID-19-negative, matched controls. The patients exhibited a significant reduction in the LnRHI at two months post-discharge compared to the controls (median = 0.55 [IQR: 0.49–0.68] vs. median = 0.70 [IQR: 0.62–0.83]; *p* = 0.012). The difference in the LnRHI between patients and controls was evident from hospitalization and persisted at two and six months without significant temporal changes. The proportion of COVID-19 patients with endothelial dysfunction (LnRHI ≤ 0.51) was 61% during hospitalization and 55% at six months. There was no significant difference in thrombotic or cardiovascular events, nor in mortality. This study demonstrates that COVID-19 adversely affects endothelial function, as evidenced by a reduction in the hyperemic vascular response index, and endothelial dysfunction may also persist.

## 1. Introduction

In December 2019, a novel single-stranded RNA virus, SARS-CoV-2, was identified in humans in Wuhan, China [1]. Except from the respiratory system, which is primarily affected, extrapulmonary complications are also encountered such as acute myocardial injury, myocarditis, thromboembolic events, and renal failure [2]. Endothelial dysfunction seems to play a major role in the pathogenesis of systemic COVID-19 involvement.

SARS-CoV-2 infection triggers endothelial cell damage directly (via virus infection) or indirectly by virus-dependent activation of the inflammatory response (via cytokine storm). SARS-CoV-2 targets the endothelium by binding to host cell receptors of angiotensin-converting enzyme 2 (ACE2). The entry into the cells’ cytoplasm is facilitated by the spike (S) protein of the virus. The activation of the S protein is assisted by type-II transmembrane serine protease 2 (TMPRSS2) and metallopeptidase domain 17 (ADAM17) [3]. The virus enters the endothelial cells through the membrane receptor ACE2, resulting in ACE2 downregulation, thereby mitigating the ACE-ACE2-Ang II-Ang 1–7-Mas receptor axis and enhancing the ACE-Ang-II-AT1R axis. In COVID-19, increased activity of angiotensin-II, in contrast to Ang 1–7, leads to elevated production of pro-inflammatory cytokines (such as IL-6, TNF alpha, and TGF beta) and pro-thrombotic effects due to nitric oxide (NO) reduction and prostacyclin release. ACE2R deficiency in the elderly and in patients with diabetes, hypertension, and heart failure who present with SARS-CoV-2 infection may account for the more severe clinical manifestations and higher number of vascular events in these patients [4]. SARS-CoV-2 infection is characterized by increased blood levels of several cytokines, including IL-6, IL-1β, IL-7, and IL-10. Epithelial and endothelial damage during initial rapid viral replication triggers exaggerated pro-inflammatory cytokine release and further enhancement of inflammation mediated by the failure of the renin–angiotensin system with loss of ACE2 function [5].

Severe SARS-CoV-2 respiratory infection results in increased reactive oxygen species synthesis and decreased NO synthesis. NO plays a crucial role in the homeostasis of the vascular endothelium. In the context of COVID-19 infection, diminished NO bioavailability exerts multifaceted effects on vascular endothelial function, including alterations in membrane permeability, upregulation of leukocyte adhesion molecules, enhanced platelet aggregation, heightened tissue oxidation, activation of thrombogenic factors, and the initiation of endothelial cell apoptosis, all of which contribute to impaired endothelial repair mechanisms [6]. Several clinical methods have been proposed for clinical assessment of endothelial function. The EndoPAT (EndoPAT2000 system (Itamar Medical, Caesarea, Israel)) is a non-operator-dependent device that is used to assess endothelial vasodilator function in a rapid and non-invasive way. The device uses a pair of novel modified plethysmographic probes, which are positioned on the index finger of each hand, detecting endothelium-mediated changes in the digital pulse waveform known as the peripheral arterial tone (PAT) signal [7].

The aim of this study is to evaluate the impact of COVID-19 infection on endothelial function during the acute phase and in the mid-term using peripheral arterial tonometry (EndoPAT).

## 2. Materials and Methods

This was a prospective cohort study with matched groups which was conducted by the Department of Cardiology in collaboration with the Department of Pulmonology and the COVID-19 Clinic of the General University Hospital of Patras, Greece, from March 2022 to January 2023.

The study included 2 groups of adult participants. Group 1 included patients who developed COVID-19 disease requiring hospitalization, while Group 2 was the control group in which COVID-19-negative volunteers were enrolled. All volunteers in the control group were asymptomatic and screened for COVID-19 with a PCR test prior to enrollment and before each visit. The two groups were matched for age, sex, and cardiovascular risk factors.

In the context of this study, the evaluation of participants’ endothelial function was performed non-invasively using the EndoPAT system (Itamar Medical, Caesarea, Israel). The EndoPAT device records endothelium-mediated changes in vascular tone after occlusion of the brachial artery. These changes reflect a downstream hyperemic response and constitute a measure for arterial endothelial function. Measurements on the contralateral arm are used to control for concurrent non-endothelium-dependent changes in vascular tone. Reactive hyperemia index (RHI) measured by EndoPAT is significantly decreased in patients with coronary artery disease or patients with one or more cardiovascular risk factors including hypertension, hyperlipidemia, diabetes, glucose intolerance, or smoking [8,9,10].

The EndoPAT system recorded the endothelium-dependent change in arterial tone. During the examination, plethysmographic biosensor probes were placed on the patient’s right and left index fingers, covering the distal 2/3 of them. Afterwards, vascular tone was measured in three phases: at rest (baseline); during ischemia induced by a cuff inflated on one of the two arms for a period of 5 min (occlusion phase); and during the phase of reactive hyperemia, after the cuff deflation and the release of the arterial blockage. Occlusion of the brachial artery was performed preferentially on the nondominant upper arm. The occlusion pressure was at least 50 mmHg above the systolic blood pressure. Each recording consisted of 5 min of baseline measurement, 5 min of occlusion measurement, and 5 min post-occlusion measurement (hyperemic period).

The participants were in the supine position for a minimum of 20 min before measurements, in a quiet, temperature-controlled (21–24 °C) room with dimmed lights. They were asked to remain silent and as immobilized as possible during the entire measurement period. Furthermore, patients were fasting for the previous 6 h.

The arterial tone signals detected in the above phases of the examination by the plethysmographic biosensor probes were converted into digital signals and the EndoPAT system software finally calculated the hyperemic vascular response (natural logarithmic reactive hyperemia index—LnRHI). Endothelial dysfunction was defined as LnRHI ≤ 0.51. No heart variability measurements were used.

The EndoPAT measurements were performed during visit 1, which referred to the hospitalization period for the COVID-19 patients and the baseline visit for the control group; visit 2, referring to the 2-month follow-up; and visit-3k, referring to the 6-month follow-up for both groups. Blood tests, i.e., blood count, C-reactive protein (CRP), D-dimers, and troponin, were collected, and electrocardiograms were performed in every hospitalized patient during the hospital stay. Cardiac events (arrhythmias, acute myocardial infarction, myocarditis, and cardiorespiratory arrest) and other complications (deep venous thrombosis, pulmonary thromboembolism, stroke, acute respiratory stress syndrome, vascular events, convulsions, pneumothorax, and pleural effusion) were recorded, as were the need for oxygen therapy or invasive mechanical ventilation, transference to the intensive care unit, and hospitalization duration. Transthoracic echocardiography was performed on the first visit day.

The World Health Organization (WHO) definition for the clinical characterization of COVID-19 disease severity was employed, with severe disease referring to patients that required oxygen support. Furthermore, critical disease describes patients who manifested complications, such as respiratory failure, acute respiratory distress syndrome, sepsis and septic shock, thromboembolism, and/or multi-organ failure (including acute kidney injury and cardiac injury) [11]. The 4C mortality score was calculated in the hospitalized patients as an index of in-hospital mortality risk [12]. According to this, four risk groups were defined with corresponding mortality rates: low risk (0–3 score, mortality rate 1.2%), intermediate risk (4–8 score, 9.9%), high risk (9–14, 31.4%), and very high risk (≥15, 61.5%) [12].

### Statistical Analysis

We used a hierarchical linear mixed-effects model to account for the repeated measurements within each subject (level 1) and 1-to-1 matching between COVID-19 and non-COVID-19 participants, based on age, sex, and cardiovascular risk (level 2). The model included fixed effects for time and group (COVID-19 vs. COVID-19-negative) and a random intercept for each (a) patient and (b) control group. An interaction between the fixed effects was included in order to investigate variation in the COVID-19 effect on LnRHI at the different time points. All analyses were carried out with the lme4 package in R (version 4.2.2) and model fit was assessed with the Akaike information criterion (AIC) and the Bayesian information criterion (BIC). An unstructured covariance matrix was used, based on the log-likelihood ratio test.

## 3. Results

### 3.1. Baseline Characteristics

This study included 23 patients hospitalized for COVID-19 and 22 matched controls. The median age was 63 years for COVID-19 patients and 65 years for the controls, and approximately one-third of patients in both groups were female. Nearly 40% of both groups were active smokers and more than 50% of both groups had hypertension. No major differences were noticed regarding baseline characteristics between the two groups (Table 1). All patients in the COVID-19 group required oxygen support during their hospitalization, and the 4C score of the affected patients had a median of 9, representing high-risk patients. The mean duration of hospital stay was 5 days (IQR: 4–9) for the enrolled COVID-19 patients.

### 3.2. Primary Endpoint and Secondary Outcomes

**Figure 1:**
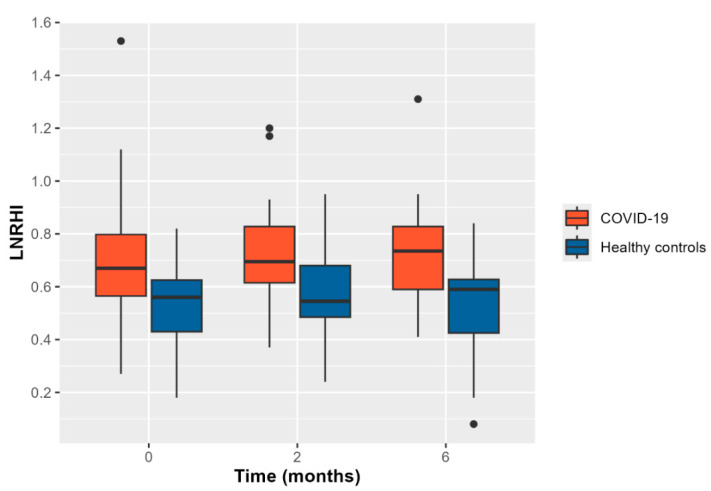
The natural logarithmic reactive hyperemia index (LnRHI) of patients suffering from COVID-19 (red box plot) and the controls (blue box plot) during hospitalization/baseline visit at 2-month and 6-month follow-up. The box represents the middle 50% of values (IQR), the horizontal line within the box represents the median, the ends of the box represent the 25th and 75th percentiles, the whiskers correspond to values ± 1.5 times the IQR on each side, and the dots represent outlier values that deviate more than 1.5 times from the IQR around the median.

Hospitalized COVID-19 patients demonstrated significantly impaired LnRHI values compared to the matched controls at the 2-month follow-up visit (0.55 in the COVID-19 group vs. 0.70 in the control group, *p* = 0.012). Furthermore, the diminished LnRHI values in the COVID-19 group were evident at visit 1, as well as at visit 3. The persisting difference in the LnRHI between the two groups is depicted in Figure 1.

**Figure 2:**
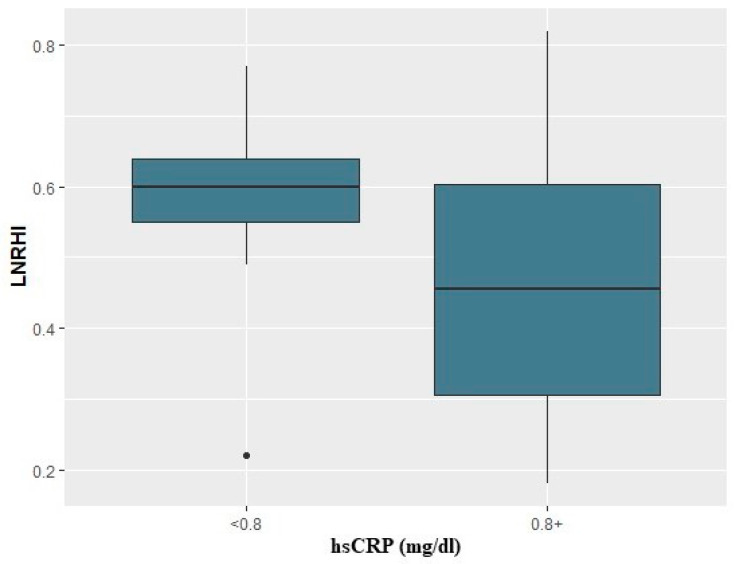
Box plot representing the relationship between hsCRP values and LnRHI in the acute setting in hospitalized COVID-19 patients. The box represents the middle 50% of values (IQR), the horizontal line within the box represents the median, the ends of the box represent the 25th and 75th percentiles, the whiskers correspond to values ± 1.5 times the IQR on each side, and the markers correspond to outliers (values outside the whiskers).

**Figure 3:**
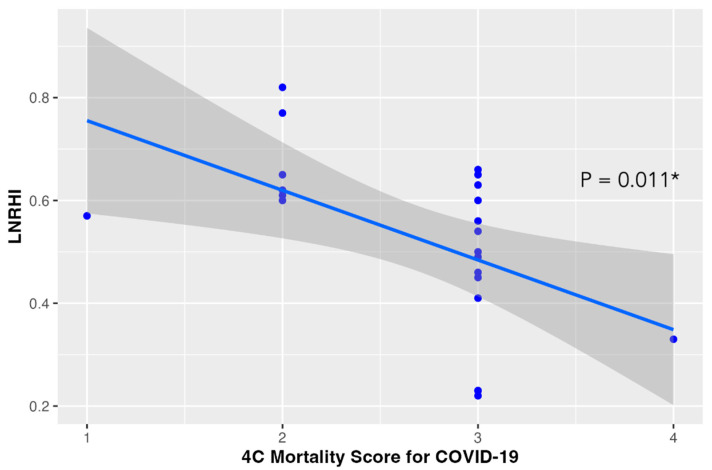
4C Mortality Score for COVID-19 and LnRHI. Higher 4C mortality score was associated with lower LnRHI values. Blue line represents the fitted linear regression with 95% confidence interval shown in the shaded area; blue dots represent individual measurements; *: *p*-value for linear trend.

Additionally, among the COVID-19 group, the observed proportion of patients with abnormal LnRHI (≤0.51) remained relatively constant across all visits. In particular, during visit 1, 14 out of 23 COVID-19 patients (61%) presented abnormal LnRHI values. Similarly, 55% of the enrolled patients presented endothelial dysfunction during 2- and 6-month follow-up. All patients enrolled were asymptomatic at 6-month follow-up. The main study outcomes are presented in Table 2. Moreover, in logistic regression analysis, baseline CRP values above 0.8 mg/dl in COVID-19 patients were associated with a 9-fold risk for compromised endothelial function (i.e., LnRHI values below 0.51) during the acute phase (OR: 9.0; 95% CI: 1.47–81.4, *p* = 0.027) (Figure 2). Finally, higher 4C mortality score was associated with lower LnRHI values and, thus, worse endothelial function (Figure 3).

## 4. Discussion

Our findings further corroborate that COVID-19 disease causes a disturbance in endothelial-mediated vascular tone. In particular, we demonstrated that COVID-19 hospitalized patients presented reduced LnRHI compared to the controls. More than half of the COVID-19 patients enrolled presented endothelial dysfunction which did not improve during the 6-month follow-up.

There is increasing evidence of endothelial dysfunction caused both directly, by the SARS-CoV-2 virus attacking the vascular endothelium (endotheliitis) [13], and indirectly, by the systemic inflammatory response and cytokine cascade. Endothelial dysfunction may contribute to the accumulation of leukocytes and the induction of tissue damage, as well as the further release of cytokines (IL-6, IL-1B, and TNF-alpha). The systemic inflammatory response disrupts the delicate balance between the pro-thrombotic and anticoagulant properties of the endothelium, leading to an increased risk of both arterial and venous thrombosis [14]. Indeed, patients with severe COVID-19 frequently suffer from pulmonary and systemic vascular complications, including pulmonary embolism, deep vein thrombosis, and major cardiovascular events. Furthermore, emerging evidence suggests that mechanisms such as apoptosis and autophagy play critical roles in endothelial cell injury, particularly in the pulmonary microvasculature, during both acute and post-acute phases of the disease [15]. These processes exacerbate endothelial damage, highlighting the complexity of the vascular involvement in COVID-19 pathology.

Our study’s results are in line with previous studies investigating the in vivo evidence of endothelial dysfunction in COVID-19 patients using an EndoPAT device [16,17]. In particular, in the study of Mejia-Renteria et al., a total of 144 patients (72 COVID-19 and 72 matched controls) were tested and LnRHI was found to be lower in the group of COVID-19 patients, especially in the subgroup of patients with past but not acute COVID-19 infection, in whom the lowest values of LnRHI were recorded (0.53 ± 0.23) [16]. This finding slightly diverges from our experience, as our data demonstrated that the endothelial impairment is present from the onset of the COVID-19 infection persisting over the 6-month study duration. A possible explanation is that our patients were hospitalized due to the infection and, thus, had more severe disease and perhaps more intense inflammatory response that could lead to earlier and more prominent endothelial dysfunction. An alternative explanation could be that a severe clinical course of COVID-19 disease is primarily associated with pathological vascular responses, resulting in a prolonged pathological vascular function in this group of patients. The former is also supported by the study of Mohammad et al., which demonstrated that LnRHI in hospitalized COVID-19 patients was significantly lower compared to non-hospitalized patients at the 3- and 6-month follow-up, revealing poorer endothelial function with more severe disease [17]. Furthermore, Gouzi et al., who studied hospitalized COVID-19 patients, found that RHI was significantly diminished in the acute phase of the disease compared to in the controls, whereas at the 4-month follow-up, there was a 51% increase in RHI. However, the authors note that COVID-19 patients with an increase in RHI had less severe systemic inflammation at baseline (reduced CRP and lower blood leukocytes and neutrophils) [18]. Finally, Cimino et al. showed that four out of six patients suffering from COVID-19 infection had impaired RHI values, median 1.32 (1.13–1.56), with normal RHI values above 1.67 [19].

Impaired endothelial function in COVID-19 has also been investigated using flow-mediated dilatation (FMD). A metanalysis by Ambrosino et al. including 12 studies is consistent with the results of our study. A total of 644 COVID-19 patients showed significantly lower FMD values as compared to 662 controls and the impaired endothelial function persisted in convalescent COVID-19 patients for up to 1 year after infection, especially when residual clinical manifestations were prominent [20].

It should be emphasized that our study included only patients who needed hospitalization but did not suffer critical illness [11]. No patients who needed intubation or admission to an intensive care unit (ICU) were included. This could justify the low percentage of adverse clinical outcomes during the follow-up period. However, in this subgroup of patients we found that in the cases with CRP values > 0.8mg/dl, there was a 9-fold risk for the occurrence of endothelial dysfunction (LnRHI ≤ 0.51). The results of other studies regarding the association of disease severity and the appearance of endothelial dysfunction are heterogeneous. In the study by Economou et al., patients hospitalized in the ICU presented lower FMD values compared to those treated in the medical ward, indicating that COVID-19 disease severity may be a significant predictor of endothelial function impairment. At the 6-month follow-up, the FMD improved but remained diminished in the COVID-19 group; however, the improvement was more prominent in patients with lower initial values (ICU patients) and independent of inflammatory biomarker levels [21]. In another study by Ambrosino et al. including 113 convalescent COVID-19 patients and 133 matched controls, although a significantly lower FMD was documented in convalescent COVID-19 patients as compared to controls, no difference in FMD values was observed between severe and critically ill patients, and no correlation was observed between FMD and the length of in-hospital stay [22]. Finally, in a study by Riou et al., impaired FMD persisting even three months after hospitalization was not associated with severe or critical SARS-CoV-2 infection, reflected by ICU hospitalization, total hospitalization duration, or severity of lung damage [23]. However, this study included only 11 ICU patients. The observed divergence may reflect differences in the documentation of disease severity or the hospitals’ protocols that may have altered during the stages of the pandemic, as well as differences in baseline characteristics, including age and cardiovascular risk factors, across the studies’ populations.

### Limitations

Some potential limitations of our study should be addressed. Our study is referring to the acute and mid-term endothelial dysfunction detected in the hospitalized COVID-19 patients. However, longer and larger follow-up studies are needed to clarify the prognostic role of LnRHI values among COVID-19 patients. Furthermore, our protocol did not include measurement of endothelial biomarkers which mediate endothelial damage and clinical sequela. Fogarty et al. recently demonstrated by utilizing blood biomarkers that sustained endotheliopathy may be involved in long COVID pathogenesis [24]. Diminished LnRHI values alongside altered endothelial biomarkers may elucidate a plausible correlation between endothelial dysfunction and long COVID.

## 5. Conclusions

The present study offers further evidence supporting the notion that COVID-19 infection negatively impacts endothelial function, as demonstrated by a reduction in the hyperemic vascular response index. Abnormal values of LnRHI were detected in more than half of the patients, persisting at the 6-month follow-up. Longer and larger studies combining PAT assessment with laboratory and clinical patient data may further elucidate the association between endothelial dysfunction and long COVID syndrome.

## Figures and Tables

**Table 1:** Baseline characteristics of COVID-19 hospitalized patients and controls.

	COVID-19 N = 23	Control N = 22	* *p*-Value
Age (years)	65 (57–74)	63 (55–72)	0.90
Female	7 (30%)	7 (32%)	1
Height (cm)	172 (164–178)	178 (166–181)	0.12
Weight (kg)	82 (70–101)	87 (71–91)	0.40
Active smoker	10 (43%)	8 (36%)	0.60
Hypertension	14 (61%)	12 (55%)	0.70
Dyslipidemia	11 (48%)	11 (50%)	0.90
Diabetes mellitus	8 (35%)	2 (9.1%)	0.071
Atrial fibrillation	2 (8.7%)	0 (0%)	0.50
Chronic kidney disease	1 (4.3%)	2 (9.1%)	0.60
Chronic medical treatment			
ASA	2 (8.7%)	3 (14%)	0.70
P2Y12i	1 (4.3%)	0 (0%)	>0.90
NOAC	4 (17%)	0 (0%)	0.11
Warfarin	1 (4.3%)	0 (0%)	>0.90
B-Blockers	5 (22%)	7 (41%)	0.40
ACEI/ARB	10 (43.5%)	9 (18%)	0.65
MRA	1 (4.3%)	0 (0%)	>0.90
Diuretics	6 (26%)	2 (9.1%)	0.20
Statins	11 (48%)	11 (50%)	0.90
Antidiabetics	8 (35%)	2 (9.1%)	0.071

* Wilcoxon rank sum test; Pearson’s Chi-squared test; Fisher’s exact test. Values are presented as median [IQR (Q1–Q3)] or n (%). ACEI: angiotensin-converting enzyme inhibitor; ARB: angiotensin receptor blocker; ASA: acetylsalicylic acid; MRA: mineralocorticoid receptor antagonist; NOAC: novel oral anticoagulant; P2Y12i: P2Y12 inhibitor.

**Table 2:** Primary and secondary endpoints during the assessment of endothelial function in hospitalized patients with COVID-19 compared to controls.

	COVID-19 N = 23	Control N = 22	* *p*-Value
LnRHI, baseline	0.56 (0.43–0.63)	0.67 (0.57–0.80)	0.007
LnRHI, 2 months	0.55 (0.49–0.68)	0.70 (0.62–0.83)	0.012
LnRHI, 6 months	0.59 (0.43–0.63)	1.78 (1.66–1.81)	0.007
LnRHI ≤ 0.51 at baseline	14 (61)	4 (18.2)	0.074
LnRHI ≤ 0.51 at 2 months	12 (55)	3 (13.6)	0.051
LnRHI ≤ 0.51 at 6 months	12 (55)	3 (13.6)	0.051
Thrombotic complications	2 (8.7)	0	0.48
Cardiovascular events	0	0	-
Deaths	0	0	-

* Wilcoxon rank sum test; Pearson’s Chi-squared test; Fisher’s exact test. Values are presented as median [IQR (Q1–Q3)] or n (%). LnRHI: Ln reactive hyperemia index.

## Data Availability

Data are available from the corresponding author upon reasonable request.
